# Silver-Protein Nanocomposites as Antimicrobial Agents

**DOI:** 10.3390/nano11113006

**Published:** 2021-11-09

**Authors:** Mahmoud Sitohy, Abdul-Raouf Al-Mohammadi, Ali Osman, Seham Abdel-Shafi, Nashwa El-Gazzar, Sara Hamdi, Sameh H. Ismail, Gamal Enan

**Affiliations:** 1 Biochemistry Department, Faculty of Agriculture, Zagazig University, Zagazig 44511, Egypt; mzsitohy@hotmail.com (M.S.); aokhalil@zu.edu.eg (A.O.); 2 Department of Science, King Khalid Military Academy, P.O. Box 22140, Riyadh 11495, Saudi Arabia; almohammadi26@hotmail.com; 3 Botany and Microbiology Department, Faculty of Science, Zagazig University, Zagazig 44519, Egypt; mora_sola1212@yahoo.com (N.E.-G.); hamdysara38@yahoo.com (S.H.); 4 Faculty of Nanotechnology for Postgraduate, Cairo University, Zayed City 12588, Egypt; drsameheltayer@yahoo.com

**Keywords:** mung bean, fenugreek, seed proteins, silver nanoparticles, nanocomposite, antibacterial activity

## Abstract

The use of nanomaterials alone or in composites with proteins is a promising alternative to inhibit pathogenic bacteria. In this regard, this study used seed proteins from both fenugreek (*Trigonella foenum-graecum* L.) (FNP) and mung bean (*Viga radiate*) (MNP), with silver nanoparticles (Ag-NPs) and nanocomposites of either Ag-NPs plus FNP (Ag-FNP) or Ag-NPs plus MNP (Ag-MNP) as inhibitory agents against pathogenic bacteria. FNP and MNP were isolated from fenugreek seeds and mung bean seeds, respectively, and fractionated using Sodium Dodecyl Sulfate–Polyacrylamide Gel Electrophoresis (SDS–PAGE). Both FNP and MNP were immobilized with Ag-NPs to synthesize the nanocomposites Ag-FNP and Ag-MNP, respectively. The physicochemical characteristics of Ag-NPs and their composites with proteins were studied by X-ray Diffraction (XRD), dynamic light scattering (DLS), the zeta potential, Scanning and Transmission Electron Microscopy (SEM and TEM, respectively), Atomic Force Microscopy (AFM), and the Brunauer–Emmett–Teller isotherm (BET), elucidating their structural parameters, size distribution, size charges, size surface morphology, particle shape, dimensional forms of particles, and specific surface area, respectively. The sole proteins, Ag-NPs, and their nanocomposites inhibited pathogenic Gram-positive and Gram-negative bacteria. The inhibitory activities of both nanocomposites (Ag-FNP and Ag-MNP) were more than those obtained by either Ag-NPs or proteins (FNP, MNP). Minimum inhibitory concentrations (MICs) of Ag-FNP were very low (20 and 10 µg mL^−1^) against *Salmonella* *typhimurium* and *Pseudomonas* *aerugenosa*, respectively, but higher (162 µg mL^−1^) against *E. coli* and *Listeria* *monocytogenes*. MICs of Ag-MNP were also very low (20 µg mL^−1^) against *Staphylococcus* *aureus* but higher (325 µg mL^−1^) against *Listeria* *monocytogenes*. TEM images of *Staphylococcus* *aureus* and *Salmonella* *typhimurium*, treated with Ag-FNP and Ag-MNP, at their MIC values, showed asymmetric, wrinkled exterior surfaces, cell deformations, cell depressions, and diminished cell numbers.

## 1. Introduction

Several published studies have reported the highest incidences of pathogenic microbes in different foods [1,2,3]. Some of the microbial pathogens isolated from different sources were identified and classified as multidrug-resistant [4,5,6,7]. Further research works are required to discover innovative strategies for controlling multidrug-resistant bacteria by (i) nanomaterials [8,9,10,11], (ii) phage therapy [12], (iii) plant extracts either singly or in combination with antibiotics [13,14], (iv) probiotics [15,16,17,18], and (v) plant or animal proteins [19,20,21]. As legume proteins have shown promising inhibitions of pathogenic multidrug-resistant bacteria in vitro and in situ [22,23], the current study was an endeavor to evaluate the antibacterial activity of nanocomposites prepared from the seed proteins of either fenugreek (FNP) or mung bean (MNP) as coupled with silver nanoparticles (AgNPs). As fenugreek seed protein is a rich-legume protein with a favorable amino acid composition, it can be a potential source of bioactive agents [24,25,26], releasing upon digestion specific bioactive peptides, which can inhibit bacterial pathogens [27]. Angiotensin I-converting enzyme (ACE) inhibitors, antioxidants, and anticancer agents can be provided by the peptides constituting the mung bean protein hydrolysate [28]. Nanoparticles explicitly used as carriers of drugs or therapeutic molecules can be used as large as 100 nm in one dimension. They are made of various materials such as natural or synthetic polymers, lipids, or metals. The quicker and more efficient biological absorption of nanoparticles than larger macromolecules nominate them as excellent delivery systems materials [29,30]. Silver nanoparticles (AgNPs) have become one of the most investigated and explored nanotechnology-derived nanostructures during the past few years, having antimicrobial activities against bacteria and fungi, counteracting multidrug-resistant bacterial strains [31]. AgNPs can disturb bacterial membranes and destroy the cells, producing severe disturbances in the cell function and structure, leading to cell death [32,33]. The present study aimed to investigate the isolation, fractionation, and characterization of fenugreek seed proteins (FNP) and mung bean seed proteins (MNP), in parallel with the characterization of AgNP nanocomposites: FNP-AgNPs and MNP-AgNPs, while following their antibacterial activities.

## 2. Materials and Methods

### 2.1. Plant Materials and Chemicals

Mung bean *(Vigra radiata* L.) seeds were purchased from the Agriculture Research Center, Cairo, Egypt. Fenugreek (*Trigonella foenum-graecum* L.) seeds were purchased from the local market, 10th of Ramadan City, Sharkia Governorate, Egypt (20 km from North Cairo). They were identified by Dr. Samir Teleb, Botany and Microbiology Department, Faculty of Science, Zagazig University. All the chemicals used were provided by Sigma chemical company (Burlington, MA, USA).

### 2.2. Microorganisms

Gram-positive pathogenic bacteria such as *Staphylococcus aureus* DSM 1104 (*S. aureus*), *Streptococcus pyogenes* ATCC 018 (*S. pyogenes*), and *Listeria monocytogenes* LMG10470 (*L. monocytogenes*) were used. Gram-negative bacteria such as *Pseudomonas aeruginosa* LMG 8029 (*P. aeruginosa*), *Escherichia coli* LMG 8223 (*E. coli*), *Salmonella typhimurium* LMG 10395 (*S. typhimurium*), *Klebsiella pneumonia* ATCC 43816 (*K. pneumonia*), and *Proteus mirabilis* WPM111 (*P. mirabilis*) were also used in this study. All the indicator bacteria were provided by the Laboratory of Bacteriology, Botany, and Microbiology Department, Faculty of Science, Zagazig University, Zagazig, Egypt. They were stored in glass beads at −20 °C and subcultured into Brain Heart Infusion broth (Oxoid Wade Road, Basingstoke, Hampshire, RG24 8PW, UK) [34].

### 2.3. Extraction of Seed Proteins (FNP, MNP)

Mung bean and fenugreek seeds were ground in a grinder (Moulinex, France) and defatted with n-hexane (Sigma Chem-Company, Burlington, MA, USA) (1:10 *w*/*v*, seeds-to-solvent ratio) with constant stirring for two hours at room temperature. The slurry was defatted two more times and then filtered using cheesecloth filters (ultrafine grade, cotton-made, Gomhuria Company, Zagazig, Egypt). After drying, the defatted seeds were used for protein isolation. About 5% (*w/v*) defatted slurry was dispersed in distilled water adjusted at pH 9.0 using 0.1 N NaOH at room temperature, shaken for one hour, and centrifuged for 15 min, at 2000× *g*. In order to obtain increased yields, the extraction and centrifugation procedures were repeated on the residue. The extracts were combined, and the pH was adjusted to 4.5 with 1 N HCL to precipitate the protein. The proteins were recovered by centrifugation at 2000× *g* for 15 min followed by removing the supernatant by decantation. Crud protein was washed with distilled water, dispersed in distilled water at pH 7.5, dialyzed against distilled water for 48 h at 4 °C, and lyophilized [35]. Both fenugreek and mung bean proteins were designated FNP and MNP, respectively, and were used in the experiments.

### 2.4. Sodium Dodecyl Sulfate–Polyacrylamide Gel Electrophoresis (SDS–PAGE)

Twenty milligrams of either MNP or FNP were dissolved in 1 mL aliquots of SDS (10%) with 100 μL of β-mercaptoethanol and subjected to intermittent vortexing for 15 min. The mixture was then centrifuged at 10,000× *g* for 5 min to separate the extract. Twenty milliliters of the extract was mixed with 20 μL of SDS sample loading buffer (SDS 4%, β-mercaptoethanol 3%, glycerol 20%, Tris-HCl 50 mM pH 6.8, and bromophenol blue traces) and heated at 96 °C for 5 min, and 10 μL aliquots were electrophoresed (10 μL of protein/lane) and analyzed by SDS–PAGE [36]. In addition, the composition of mung bean and fenugreek seed proteins was investigated by Powder X-ray Diffraction (XRD) (Bruker, D8 discover).

### 2.5. Synthesis of Silver Nanoparticles (AgNPs)

The synthesis of silver nanoparticles was achieved following the co-precipitation method, employing tri-sodium citrate (TSC) as a reducing and capping agent. First, AgNO_3_ solution (0.02 M) was dissolved in 100 mL of deionized water, heated to boiling, and then TSC was added drop by drop with rough stirring (750 rpm) and heated until the mixture color became pale yellow. Finally, the mixture was cooled to room temperature under dark conditions to avoid light [37].

### 2.6. Synthesis of FNP and MNP Nanoparticles

Synthesis of FNP (fenugreek protein nanoparticles) and MNP (mung bean protein nanoparticle) was carried out by the top-down method in which the large particles (bulk) were converted to small ones (nanoparticles). Both fenugreek and mung bean, extracted proteins, used as source materials, were of more than 98.5% and 99.8% purity, respectively. The ball mill method was used to prepare either FNP or MNP with multi-step processes. A quantity (5 g) of either fenugreek or mung bean was charged into 40 cm ball milling stainless-steel vials; ball mills consisted of silicon carbide and stainless-steel balls that were mounted on a vibrating plate. First, 10 g of stainless-steel balls with a 0.2 cm diameter were added to the vibrating plate, and the milling was conducted for 10 h. Secondly, the silicon carbide balls with a diameter of 0.02 cm were added to the vibrating plate, and milling lasted for 10 h [37].

### 2.7. Synthesis of Silver-Fenugreek Nanocomposite (Ag-FNP) or Silver-Mung-Bean Nanocomposite (Ag-MNP)

Synthesis of either the silver-fenugreek nanocomposite (Ag-FNP) or silver-mung-bean nanocomposite (Ag-MNP) was carried out by direct precipitation of silver nanoparticles in the presence of fenugreek and mung bean nanoparticles through a synthesis process. First, 1 g of mung bean or fenugreek dispersion in 200 mL of deionized distilled water was added to a 250 mL beaker of 0.1 g of silver nitrate and heated until boiling. Then, trisodium citrate (5 g/50 mL) was added dropwise under stirring at 800 rpm until the yellow color appeared.

### 2.8. Characterization of Both AgNPs and Nanocomposites (Ag-FNP, Ag-MNP)

XRD (Bruker, D8 discover) (Billerica, MA, USA) was applied to Ag-FNP and Ag-MNP to confirm their colloidal nature and to test the homogeneity and purity of synthesis processes. In addition, the size and charge of both Ag-FNP and Ag-MNP were measured by both dynamic light scattering (DLS) and zeta potential (Entgris, Z3000) (Billerica, MA, USA), as previously described in [38,39,40].

Scanning Electron Microscopy (SEM) (JEOL, Akishima, Tokyo 196-8558, Japan) was carried out also to study the surface morphology of AgNPs, Ag-FNP, and Ag-MNP. According to Jol 2000, Japan, the SEM images were taken, operating at an acceleration voltage of 20 kV and magnification of 160,000×. In addition, Transmission Electron Microscopy (TEM) was carried out for both AgNPs and either Ag-FNP or Ag-MNP. Either AgPNs or Ag-MNP and Ag-FNP were added to double-deionized water and sonicated for 50 min using an ultrasound instrument of 50 kHz, at an amplitude of 85% and 0.65 of a cycle (UP400S, Hielscher, Germany). An aliquot (5 microns) of the slurry was then placed onto a carbon-coated copper grid. TEM examinations were carried out using a TEM-2100 high-resolution electron microscope (JEOL, Akishima, Tokyo 196-8558, Japan).

Atomic Force Microscopy (AFM 5600LS, Agilent, Santa Clara, California, USA) was used to provide 2-dimensional and 3-dimensional AFM of both AgNPs and either Ag-FNP and Ag-MNP. First, samples were subjected to ultrasound waves for one hour, a condition of 60 kHz, and an amplitude of 85% and 0.6 of a cycle (UP400S manufactured by Hielscher, Teltow, Germany); then, a thin film was created using a spin coating instrument model Laurell-650Sz under the condition of 820 rpm under vacuum [41].

The specific surface area was measured for both proteins (FNP, MNP) and protein-nanocomposites (Ag-FNP, Ag-MNP) by the BET method (the Brunauer–Emmett–Teller isotherm). A Quantachrome, NOVA touch LX2 model was used in this work. Samples were degassed at 50 °C for 3 h. Nitrogen was the adsorbate model, with the following specifications: cross-sectional area (16.2 Å^2^/molec), molecular weight (28.0134 g), bath temperature (77.35 K), magnetic susceptibility (2 (mL/mol) × 10^−29^), critical pressure (33.5 atm), critical temperature (126.2 K), and supercritical adsorption. In addition, the isotherm curves of AgNPs and Ag-protein nanocomposites were constructed as described by [41].

### 2.9. Antibacterial Activity of AgNPs, Protein Nanoparticles, and Ag-Protein Nanocomposite

The antibacterial activity of AgNPs, protein only (FNP and MNP), and protein plus Ag NPs in composites (Ag-FNP and Ag-MNP) was studied against the indicator Gram-positive bacteria such as *S. aureus*, *S. pyogene,* and *L. monocytogenes* and Gram-negative bacteria such as *P. aeruginosa*, *E. coli*, *S. typhimurium*, *K. pneumonia,* and *P. mirabilis*. To assess the MIC of different substances against different bacteria, tube dilution was used. The tested substance was serially diluted in bacterial growth media, added to the test organisms, and incubated, and the bacterial growth was observed and recorded. The MIC is defined as the lowest concentration preventing observable bacterial growth on culture plates. The bacterial suspensions were spread over the surface of nutrient agar plates. Then, sterilized filter paper discs of about 6 mm in diameter were soaked in each tested material (1 MIC expressed as μg/mL). In another experiment, they were soaked in extracts of either proteins or nanocomposites at different concentrations (1300, 195 650, 325, 162, 80, 40, 20, and 10 μg/mL) and were then laid onto the surface of nutrient agar media (Oxoid) and inoculated with different tested bacteria with appropriate distances separating them from each other. The nutrient agar plates were incubated at 37 °C for 24–48 h. Diameters of inhibition zones were measured using a millimeter ruler [42,43].

### 2.10. Transmission Electron Microscopy (TEM) of Sensitive Bacteria in Response to the Proteins and Nanocomposites Used

*S. aureus* and *S. typhimurium*, selected for the TEM studies, were propagated in Brain Heart Infusion broth for 24 h at 37 °C. Cell suspensions were centrifuged at 10,000 rpm for 10 min, and the cell pellets were resuspended in buffered peptone water (0.1% peptone plus 0.85% NaCl) and diluted to 10^5^ CFU/mL as the final concentration. They were then treated with MIC values of either Ag-FNP or Ag-MNP and incubated at 37 °C for 4 h. Bacterial cells were then fixed in glutaraldehyde (2.5% in 0.1 M of phosphate buffer (pH 7.4) and post-fixed with 1% osmium tetroxide for 2 h at 4 °C. The washing step was repeated, and the cells were dehydrated sequentially using 30%, 50%, 70%, and 95% acetone for 15 min for each and finally with 100% acetone three times for 30 min. Subsequently, cells were treated with propylene oxide twice for 10 min at 4 °C and sequentially filtrated with a mixture of propylene oxide and Durcupan’s ACM epoxy resin (3:1, 1:1, and 1:3) for 45 min. Polymerization of the resin to form specimen blocks was performed in an oven at 60 °C for 72 h. The specimen blocks were sectioned with a diamond knife in a Reichert Ultracut R ultramicrotome (Leica, Wetzler, Germany). Thin sections (70–80 nm) were placed on 300 mesh copper grids, stained for 15–20 min in uranyl:ethyl alcohol (1:1), and then washed three times with saline solution for 2 min. A drop of Reynol’s lead citrate was added before examination using a TEM (JEOL, Akishima, Tokyo 196-8558, Japan) [44,45].

### 2.11. Statistical Analysis

All data were subjected to statistical analysis by the one-way ANOVA test using SPSS software for Windows version 22 (Armonk, NY, USA: IBM Corp.). A probability of *p* ≤ 0.05 was considered as the level of significance unless otherwise stated.

## 3. Results

**Figure 1:**
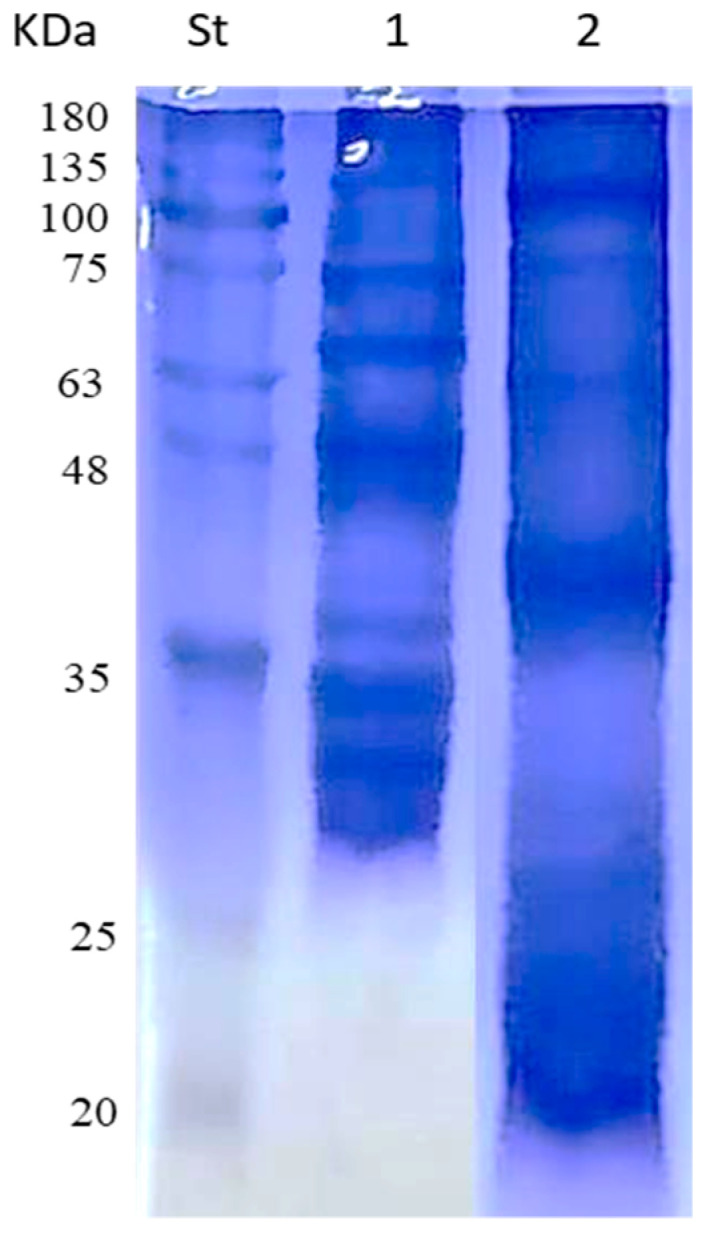
SDS–PAGE of mung bean protein (MNP) (lane 1) and fenugreek protein (FNP) (lane 2) compared to protein standard (St).

**Figure 2:**
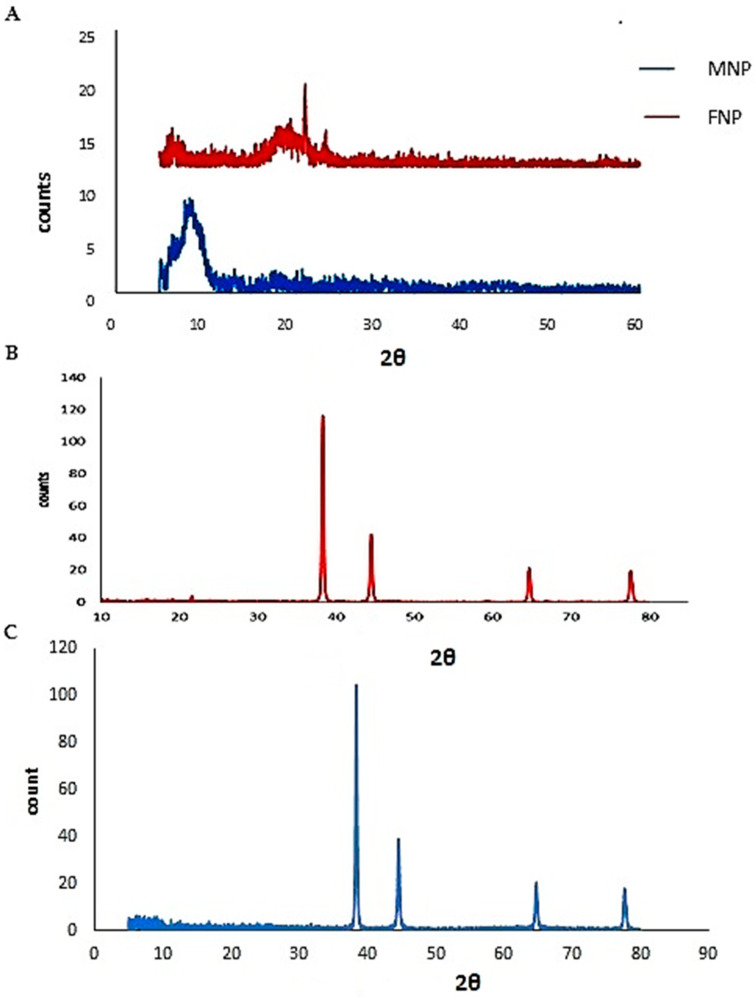
Powder X-ray Diffraction (XRD) pattern for (**A**) MNP and FNP, (**B**) Ag-FNP, and (**C**) Ag-MNP.

FNP and MNP were the outputs of fenugreek and mung bean seed protein, respectively. For characterizing FNP and MNP, Sodium Dodecyl Sulfate–Polyacrylamide Gel Electrophoresis (SDS–PAGE) was run and the results are shown in Figure 1. FNP showed five protein bands corresponding to 20, 25, 40, 63, and 100 kDa, while MNP showed seven bands corresponding to 30, 32, 40, 48, 65, 75, and 135 kDa. The outputs of X-ray Diffraction (XRD) examination of FNP and MNP are given in Figure 2. One characteristic peak at a 2θ angle of 9.1° and two characteristics peaks at 2θ angles of 21.709 and 24.001° were noticed for FNP and MNP, respectively. Combining the two proteins with AgNPs gave nanocomposites (Ag-FNP, Ag-MNP). Both Ag-FNP and Ag-MNP showed four sharp characteristic peaks at 2θ angles of 38.26, 44.47, 64.71, and 77.73°, indicating the cubic lattice of nanocomposites, distinguishing them from either FNP or MNP.

**Figure 3:**
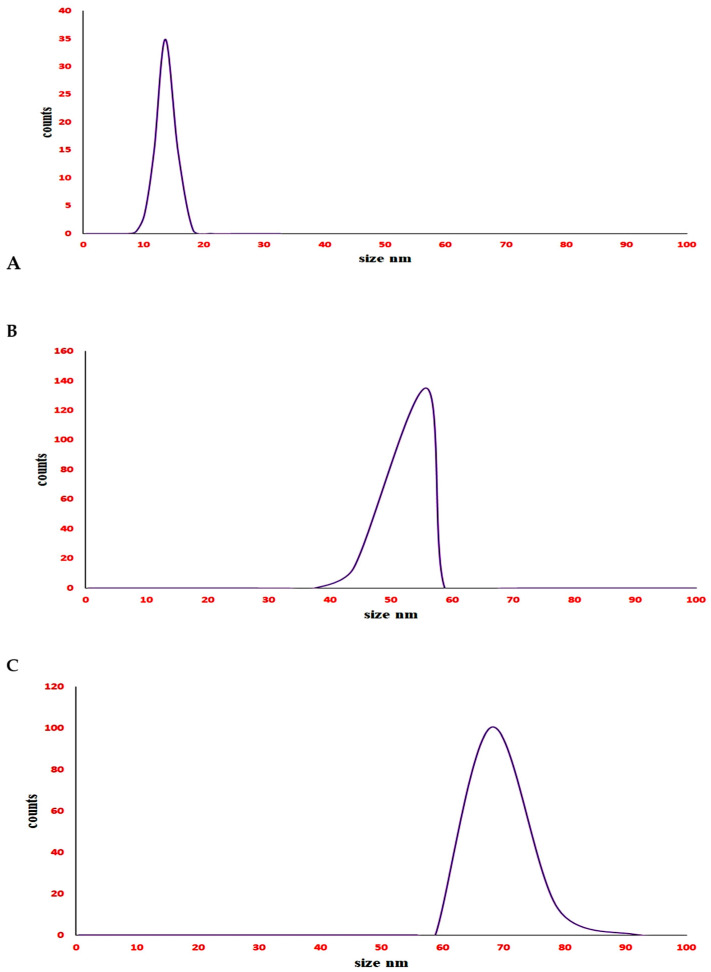
DLS for (**A**) silver nanoparticles (AgNPs); (**B**) silver mung bean protein nanocomposite (Ag-MNP), and (**C**) silver fenugreek protein nanocomposite (Ag-FNP).

**Figure 4:**
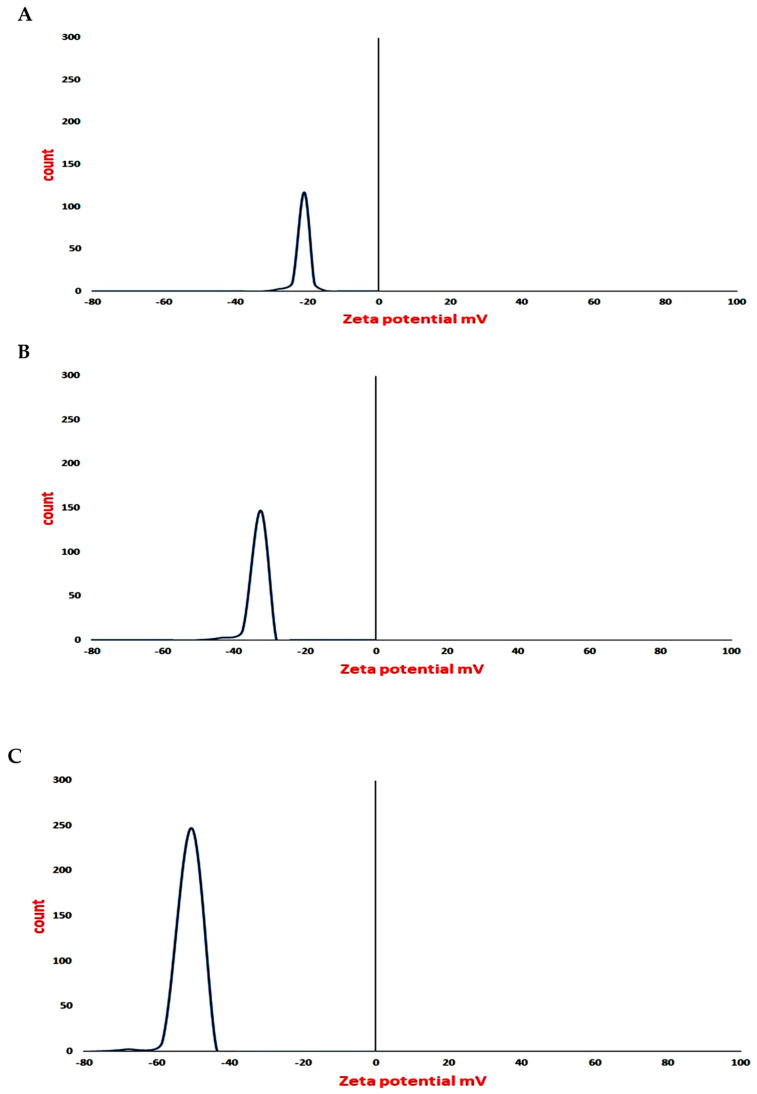
Zeta potential value for (**A**) silver nanoparticles (AgNPs), (**B**) silver mung bean protein nanocomposite (Ag-MNP), and (**C**) silver fenugreek protein nanocomposite (Ag- FNP).

The characteristic peaks of DLS analysis were at 13.55, 55.50, and 65.02 nm for AgNPs, Ag-MNP, and Ag-FNP, respectively. The appearance of one peak only for each of them indicated the homogeneity of their forms (Figure 3). The peaks at −22, −34, and −49 mV were characteristic zeta potential values for AgNPs, Ag-MNP, and Ag-FNP, respectively (Figure 4). The sharp and high peaks of zeta potential values may refer to the purity and homogeneity of each. 

**Figure 5:**
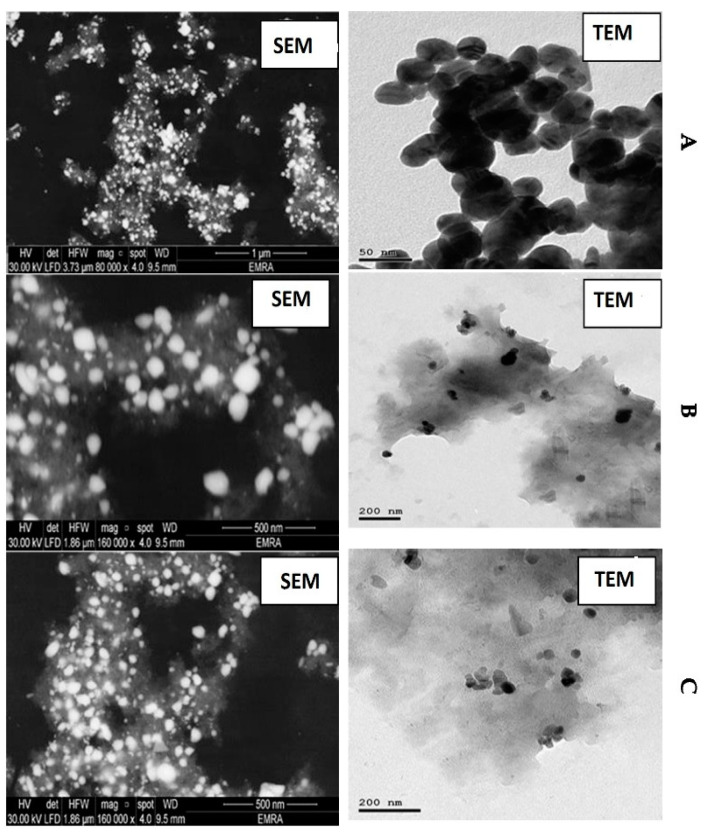
Microscopic characterization of silver-protein nanocomposites: (**A**–**C**). TEM images of three different silver-protein nanocomposite formulations showing distinct morphological features at 100 nm scale; (**A**–**C**), SEM images of the same silver nanoparticle sample imaged at progressive magnification levels (1 μm, 500 nm, and 500 nm respectively) to demonstrate the hierarchical surface morphology and aggregation patterns of silver nanoparticles. The sequential magnification allows visualization of both the overall distribution and the detailed surface features of the same nanostructure. The all SEM images for silver-mung bean seed protein nanocomposite (Ag-MNP) (Supplementary Figure S1) and silver-fenugreek seed protein nanocomposite (Ag-FNP). (Supplementary Figure S2) SEM images in supplementary file attached.

In Figure 5B, the TEM image represents transmission electron microscopy of silver-protein nanocomposite material. The background matrix textures visible in the image are characteristic of the protein matrix in which the silver nanoparticles are embedded. In transmission electron microscopy of nanocomposites, such consistent background patterns are expected due to the uniform protein matrix structure and do not indicate image manipulation. The apparent similarities in nanoparticle regions result from our controlled synthesis protocol, which was specifically designed to produce particles with uniform size distribution and morphology using highly controlled synthesis methods.

**Figure 6:**
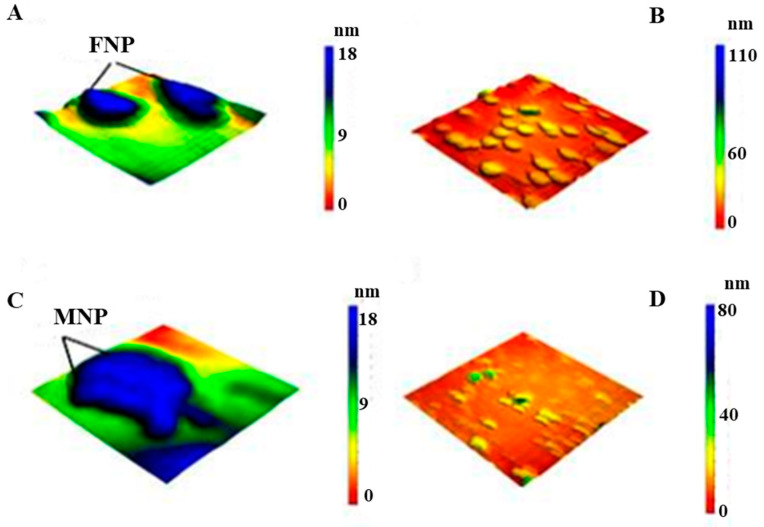
The three-dimensional form of Atomic Force Microscope (AFM) images of 50 × 50 nm of (**A**) FNP, (**B**) Ag-FNP, (**C**) MNP, and (**D**) Ag-MNP.

The AFM images consolidated the results of electron microscopic examinations wherein both FNP and Ag-FNP showed subtriangular shapes. However, both MNP and Ag-MNP showed rectangular shapes (Figure 6).

The BET surface appeared to be 102.56, 69.64, 61.65, and 45.4395 m^2^/g for FNP, Ag-FNP, MNP, and Ag-MNP, respectively (Supplementary Figure S3). The decrease in BET surface area in some samples may be due to the formation of nanocomposites with AgNPs. All the samples showed V isotherms shapes (Supplementary Figure S4).

The antibacterial activities of AgNPs, sole proteins (FNP and MNP), and protein nanocomposites (Ag-FNP, Ag-MNP) were studied against both Gram-positive and Gram-negative pathogenic bacteria (Table 1). All the tested antimicrobial agents (at MIC) showed antibacterial activity of significantly distinctive values (*p* < 0.05). Both Ag-FNP or Ag-MP showed broader antibacterial activity than those obtained by either AgNPs or proteins (FNP and MNP). *S. aureus* (Gram-positive) and *S. typhimurium* (Gram-negative) appeared as the most sensitive organisms (Table 1 and Supplementary Figure S5).

The MICs of FNP, MNP, Ag-NP, Ag-FNP, and Ag-MNP were determined against all experimental bacterial strains. For FNP, the MIC was recorded from 625 to 10,000 µg mL^−1^ and from 2500 to 5000 µg mL^−1^ in case G− and G+ bacteria, respectively (Table 2). For MNP, the MIC was recorded from 2500 to 10,000 µg mL^−1^ and from 5000 to 10,000 µg mL^−1^ in case G− and G+ bacteria, respectively. For Ag-NP, the MIC values ranged between 325 and 162 µg mL^−1^ for G+ and G−, respectively (data not shown). For Ag-FNP, the MIC ranged from 10 to 162 µg mL^−1^, while it was in the range from 20 to 325 µg mL^−1^ for Ag-MNP (Table 3).

**Figure 7:**
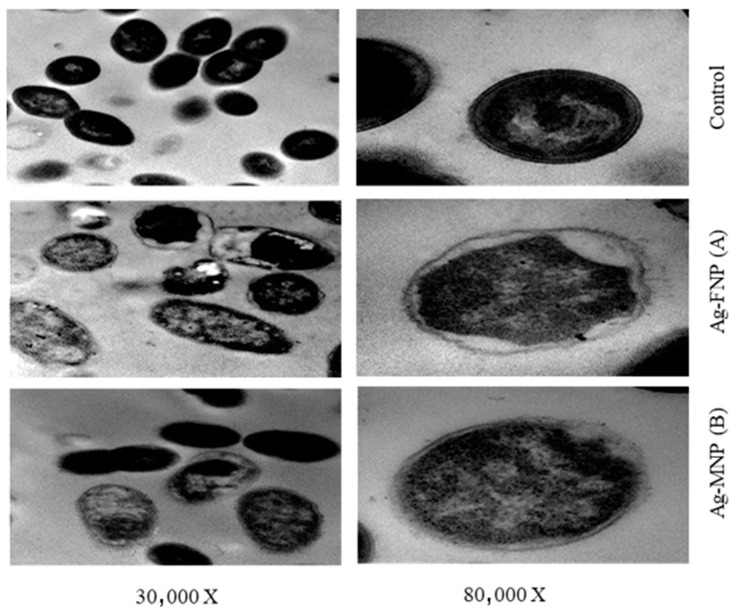
TEMs of *S. typhimurium* affected by MIC of (**A**) Ag-FNP and (**B**) Ag-MNP at (30,000×, 80,000×). MICs of Ag-FNP and Ag-MNP against *S. typhimurium* were 20 and 40 µg/mL, respectively.

**Figure 8:**
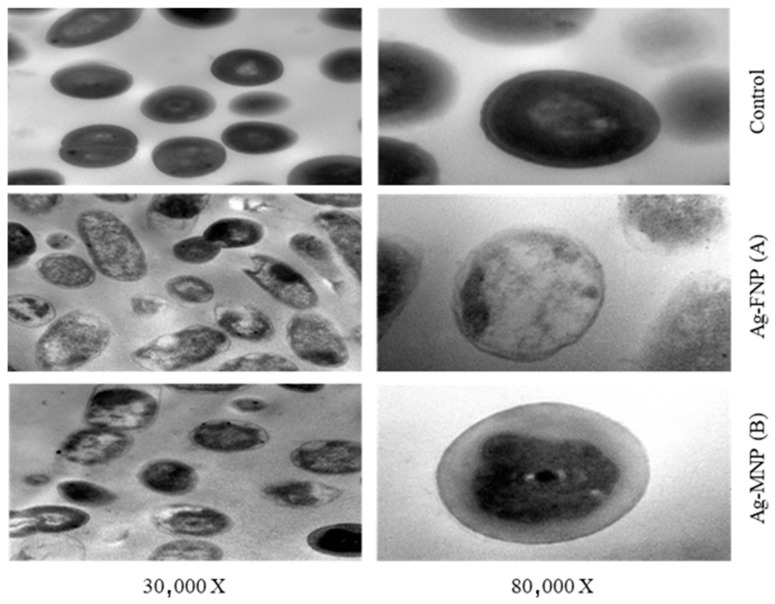
TEMs of *S. aureus* as affected by MIC of (**A**) Ag-MNP and (**B**) Ag-FNP at (30,000×, 80,000×). The MIC of Ag-FNP and Ag-MNP against *S. aureus* was 20 µg/mL.

TEM images showed the reducing effect of Ag-MNP and Ag-FNP on the relative content of the intact cells of *S. typhimurium* (OD600 = 0.5 at the time of application) after four hours of incubation at 37 °C in the nutrient broth media (Figure 7 and Figure 8). Some bacterial cells showed different manifestations of deformation. Ag-FNP and Ag-MNP induced similar signs of effects on *S. aureus* and *S. typhimurium* in nutrient broth media, including cell shrinkage, cell membrane wrinkles, pore formation, and emptiness of bacterial cells. TEM results indicated that the action of the cationic proteins targeted the cell wall and cell membrane more. Ag-FNP and Ag-MNP caused high rates of bacterial cell lysis (measured by OD600) in both *S. aureus* and *S. typhimurium.*

## 4. Discussion

The high incidence of resistant bacteria variants to antimicrobial food additives has vastly impacted human mortality and healthcare [18]. Thus, there is an urgent demand to find alternative antimicrobial food additives capable of simulating the innate immune systems. Antimicrobial peptides are highly active against most microbes, including both Gram-positive and Gram-negative bacteria [46]. Consequently, it is supposed that the peptides are less bacterial resistance-generating than other antimicrobials are [20,44]. The antimicrobial properties of plant proteins support their use as alternative food preservatives [47]. Several classes of plant proteins with antibacterial and/or antifungal properties have been isolated, identified, and recommended as antimicrobial agents [48,49,50]. Cationic antimicrobial peptides or proteins (AMPs) are still the best choices and most promising candidates for antibacterial agents [51,52] based on numerous studies indicating their broad-spectrum antimicrobial activities against Gram-positive and Gram-negative pathogenic bacteria [53,54]. The native proteins may be good antibacterial candidates, based on their amino acid compositions (FNP and MNP). The relative amounts of polar acidic, basic, and hydrophobic amino acids in FNP represent about 31.6, 14.3, and 28.5% against 33, 26.5, and 36.33% in MNP, respectively [55,56]. The antibacterial activity of the native protein may be due to its content of positively charged cationic and hydrophobic residues of amino acids [57,58,59,60]. The MICs of FNP and MNP were recorded between 5000 and 10,000 µg/mL. These low impacts of these two fractions (FNP and MNP) as antibacterial agents may be due to the high molecular weight for native proteins and the neutralization of the positively charged protein subunits by negatively charged ones. Therefore, there is a need to continue developing safe antimicrobial proteins through nanoparticles formation.

Nanoparticles are now considered viable alternatives to antibiotics and seem to have a high potential to counteract the emerging multidrug-resistant bacteria [4]. In particular, silver nanoparticles (AgNPs) have attracted much attention in the scientific field [5,13] and have always been used against various diseases. In the past, it proved its effectiveness as an antiseptic and an antimicrobial agent against Gram-positive and Gram-negative bacteria [22,23,24]. AgNPs were considered particularly attractive for producing a new class of antimicrobials [61], opening up an entirely new way to combat a wide range of bacterial pathogens based on nanomaterials [12]. The data confirmed a single peak by the DIS technique at 13, 55, and 65 nm for AgNPs, Ag-MNP, and Ag-FNP, respectively. It was concluded from SEM and TEM analysis that AgNPs were well dispersed in the solution with different shapes without any agglomeration. This fact might explain the variability of molecules that are liable for the formation of AgNPs. These molecules were used for capping and stabilizing agents and preventing AgNPs agglomeration [9]. In addition, AFM detected the subrectangular shape of the new nanocomposite with protein without any agglomeration, similar to that reported previously [9]. The present investigation by X-ray Diffraction illustrated the presence of characteristic peaks and a cubic lattice of silver nanoparticles in a new nanocomposite of mung bean and fenugreek proteins. 

The BET results showed that the area was lower in the protein nanocomposite than in the single nanoparticles. El-Gazzar and Ismail [10] recorded that the low BET surface area could be characteristic of the nanocomposite. In the current study, the nanocomposite of proteins (FNP, MNP) with AgNPs recorded a low BET surface area, confirming the latter published results [10]. The inhibitory effects of nanoparticles varied according to their size and concentration. Several studies have shown that AgNPs activity is strongly size-dependent [62]. In the current study, the strongest antibacterial activity was specifically detected against *S. aureus*. The correlation between the bactericidal effect and AgNPs concentrations is bacterial class-dependent [63]. Accumulating scientific evidence has demonstrated that AgNPs activity would depend not only on their concentration [64] but also on their shape [65].

## 5. Conclusions

Ag-FNP and Ag-MNP can be employed as competent natural inhibitors against pathogenic bacteria, e.g., *S. aureus* and *S. typhimurium*. The antimicrobial activity of the constituting proteins may be ascribed to the positively charged cationic residues of the alkaline amino acids such as arginine and lysine and the high ratio of the hydrophobic residues, e.g., leucine and valine, which can interact with the bacterial membranes, causing pores within the bacterial cell membrane, leading, finally, to their death. TEM images of *S. aureus* and *S. typhimurium* treated with Ag-FNP and Ag-MNP exhibited cell deformations, adherence to lysed cell content leading to cell clumping, malformations, blisters, and cell depressions, and diminished cell numbers. 

## Figures and Tables

**Table 1:** Antibacterial activity of (at 1 MIC) AgNPs, FNP, MNP, Ag-FNP, and Ag-MNP.

Treatment/Pathogenic Bacteria	Inhibition Zone Diameter (mm)				
	AgNPs	FNP	MNP	Ag-FNP	Ag-MNP
Gram-Negative Bacteria					
*K. pneumonia*	10.3 ± 0.3 ^j–o^	13.67 ± 0.6 ^h–l^	10.0 ± 0.7 ^j–o^	53.0 ± 0.4 ^b,c^	43.0 ± 0.5 ^d^
*S. typhiurium*	12.0 ± 0.6 ^i–n^	15.3 ± 0.5 ^h–j^	21.0 ± 0.2 ^g^	53.0 ± 0.1 ^b,c^	53.0 ± 0.5 ^b,c^
*E. coli*	14.0 ± 0.4 ^h–l^	5.67 ± 0.6 ^op^	9.0 ± 0.3 ^k–p^	43.0 ± 0.1 ^d^	50.0 ± 0.8 ^c^
*P. aerugenosa*	14.67 ± 0.5 ^h–k^	9.0 ± 0.3 ^k–p^	6.3 ± 0.8 ^n–p^	40.0 ± 0.3 ^d,e^	31.0 ± 0.1 ^f^
*P. mirabilis*	11.67 ± 0.2 ^i–n^	4.3 ± 0.7 ^p^	6.3 ± 0.1.1 ^n–p^	52.0 ± 0.3 ^b,c^	53.0 ± 0.6 ^b,c^
Gram-Positive Bacteria					
*S. pyogenes*	16.3 ± 0.3^g–i^	7.3 ± 0.6 ^m–p^	11.67 ± 1.5 ^i–n^	31.0 ± 0.4 ^f^	31.0 ± 0.3 ^f^
*S. aureus*	13.0 ± 0.6^i–m^	10.3 ± 0.1 ^j–o^	12.0 ± 0.8 ^i–n^	66.0 ± 0.2 ^a^	68.0 ± 1.0 ^a^
*L. monocytogenes*	18.67 ± 0.5 ^g,h^	11.3 ± 0.4 ^i–o^	8.67 ± 1.2 ^l–p^	35.3 ± 1.3 ^e,f^	31.0 ± 0.5 ^f^

FNP: fenugreek seed proteins, MNP: mung bean protein, AgNPs: silver nanoparticles, Ag-FNP: silver fenugreek protein nanocomposite, Ag-MNP: silver mung bean protein nanocomposite. Every value is the average of three replicates ± SE. Letters (a–p) in same column refer to significantly different values (*p* < 0.05).

**Table 2:** Minimum inhibitory concentration (MIC; µg mL^−1^) of tested proteins against Gram-positive and Gram-negative bacteria.

Treatment/Pathogenic Bacteria	MIC (µg mL^−1^)				
	AgNPs	FNP	MNP	Ag-FNP	Ag-MNP
Gram-negative bacteria					
*K. pneumonia*	162	10,000	10,000	40	162
*S. typhiurium*	162	5000	5000	20	40
*E. coli*	162	625	5000	162	162
*P. aerugenosa*	162	5000	5000	80	80
*P. mirabilis*	162	1250	2500	80	80
Gram-positive bacteria					
*S. pyogenes*	325	5000	10,000	40	162
*S. aureus*	325	5000	5000	10	20
*L. monocytogenes*	325	2500	5000	162	325

**Table 3:** Antibacterial activities of both Ag-FNP and Ag-MNP against different indicator bacterial pathogens.

Concentration (µg mL^−1^)	Inhibition Zone Diameter (mm) Against Sensitive Bacteria															
	Gram-Negative										Gram-Positive					
	*K. pneumonia*		*S. typhimurium*		*E. coli*		*P. aerugenosa*		*P. mirabilis*		*S. pyogenes*		*S. aureus*		*L. monocytogenes*	
	A	B	A	B	A	B	A	B	A	B	A	B	A	B	A	B
1300	51 ± 2.5 ^a^	43 ± 1 ^c^	53 ± 2.5 ^a^	58 ± 2 ^a^	43 ± 2 ^b^	50 ± 1 ^b^	40 ± 1.73 ^b^	30 ± 3 ^d^	51 ± 0.5 ^a^	53 ± 2 ^b^	33 ± 2 ^b^	33 ± 2 ^b^	66 ± 4 ^a^	66 ± 4 ^a^	35 ± 5 ^b^	35 ± 5 ^b^
650	32 ± 2 ^c^	31 ± 1 ^c^	45 ± 5 ^a,b^	43 ± 1 ^b^	31 ± 2 ^c^	39 ± 4 ^a^	40 ± 2 ^b^	46 ± 4 ^a,b^	49 ± 2 ^a^	50 ± 5 ^a^	33 ± 2 ^b^	20 ± 2 ^a^	66 ± 4 ^a^	50 ± 5 ^b^	35 ± 5 ^b^	22 ± 3 ^c^
325	22 ± 2 ^c^	21 ± 1 ^b^	34 ± 1 ^b^	30 ± 3 ^c^	25 ± 5 ^c^	31 ± 2 ^c^	35 ± 2 ^b^	34 ± 1 ^b^	45 ± 2 ^a^	43 ± 2 ^a^	27 ± 2 ^b^	11 ± 2 ^b^	50 ± 2 ^a^	42 ± 3 ^b^	20 ± 2 ^b^	11 ± 1 ^c^
162	17 ± 3 ^c^	15 ± 1 ^c^	30 ± 2 ^b^	25 ± 2 ^b^	17 ± 1 ^c^	15 ± 1 ^c^	29 ± 2 ^b^	22 ± 2 ^b^	39 ± 3 ^a^	25 ± 2 ^b^	22 ± 3 ^a^	8 ± 1 ^a^	41 ± 3 ^a^	31 ± 1 ^a^	12 ± 1 ^b^	0 ± 0 ^b^
80	12 ± 3 ^c^	0 ± 0 ^b^	25 ± 2 ^a^	17 ± 2 ^a^	0 ± 0 ^d^	0 ± 0 ^b^	25 ± 1 ^a^	15 ± 3 ^a^	21 ± 2 ^b^	16 ± 1 ^a^	15 ± 1 ^a^	0 ± 0 ^b^	30 ± 1 ^a^	25 ± 5 ^a^	0 ± 0 ^c^	0 ± 0 ^b^
40	9 ± 1 ^c^	0 ± 0 ^b^	15 ± 2 ^b^	12 ± 1 ^b^	0 ± 0 ^d^	0 ± 0 ^b^	20 ± 2 ^a^	0 ± 0 ^b^	0 ± 0 ^d^	0 ± 0 ^b^	9 ± 1 ^a^	0 ± 0 ^b^	21 ± 2 ^a^	19 ± 3 ^a^	0 ± 0 ^b^	0 ± 0 ^b^
20	0 ± 0 ^c^	0 ± 0 ^b^	9 ± 2 ^b^	0 ± 0 ^b^	0 ± 0 ^c^	0 ± 0 ^b^	16 ± 1 ^a^	0 ± 0 ^b^	0 ± 0 ^c^	0 ± 0 ^b^	0 ± 0 ^b^	0 ± 0 ^b^	13 ± 2 ^a^	11 ± 3 ^a^	0 ± 0 ^b^	0 ± 0 ^b^
10	0 ± 0 ^b^	0 ± 0 ^b^	0 ± 0 ^b^	0 ± 0 ^b^	0 ± 0 ^b^	0 ± 0 ^b^	9 ± 2 ^a^	0 ± 0 ^b^	0 ± 0 ^b^	0 ± 0 ^b^	0 ± 0 ^b^	0 ± 0 ^b^	0 ± 0 ^b^	0 ± 0 ^b^	0 ± 0 ^b^	0 ± 0 ^b^
5	0 ± 0 ^b^	0 ± 0 ^b^	0 ± 0 ^b^	0 ± 0 ^b^	0 ± 0 ^b^	0 ± 0 ^b^	0 ± 0 ^b^	0 ± 0 ^b^	0 ± 0 ^b^	0 ± 0 ^b^	0 ± 0 ^b^	0 ± 0 ^b^	0 ± 0 ^b^	0 ± 0 ^b^	0 ± 0 ^b^	1 ± 0 ^b^

(**A**) Ag-FNP: silver fenugreek protein nanocomposite, (**B**) Ag-MNP: silver mung bean protein nanocomposite. Every value is the average of three replicates ± SE. Letters (a–d) in same row refer to significantly different values (*p* < 0.05).

## Data Availability

Data are available on request.

## Supplementary Materials

The following are available online at https://www.mdpi.com/article/10.3390/nano11113006/s1, Supplementary Figure S1. Silver-mung bean seed protein nanocomposite (Ag-MNP) SEM images; Supplementary Figure S2. Silver-fenugreek seed protein nanocomposite (Ag-FNP) SEM images; Supplementary Figure S3. The specific surface area measured with the Brunauer–Emmett–Teller isotherm (BET) of (A) FNP; (B) Ag-FNP; (C) MNP; (D) Ag-MNP; Supplementary Figure S4. The isotherm curve of (A) FNP; (B) Ag-FNP; (C) MNP; (D) Ag-MNP; Supplementary Figure S5. Antibacterial activity of silver mung bean protein nanocomposite (Ag-MNP), and silver fenugreek protein nanocomposite (Ag-FNP) against Gram-positive and Gram-negative bacteria.
