# Correction: Sitohy et al. Silver-Protein Nanocomposites as Antimicrobial Agents. *Nanomaterials* 2021, *11*, 3006

**DOI:** 10.3390/nano15241848

**Published:** 2025-12-09

**Authors:** Mahmoud Sitohy, Abdul-Raouf Al-Mohammadi, Ali Osman, Seham Abdel-Shafi, Nashwa El-Gazzar, Sara Hamdi, Sameh H. Ismail, Gamal Enan

**Affiliations:** 1 Biochemistry Department, Faculty of Agriculture, Zagazig University, Zagazig 44511, Egypt; mzsitohy@hotmail.com (M.S.); aokhalil@zu.edu.eg (A.O.); 2 Department of Science, King Khalid Military Academy, P.O. Box 22140, Riyadh 11495, Saudi Arabia; almohammadi26@hotmail.com; 3 Botany and Microbiology Department, Faculty of Science, Zagazig University, Zagazig 44519, Egypt; mora_sola1212@yahoo.com (N.E.-G.); hamdysara38@yahoo.com (S.H.); 4 Faculty of Nanotechnology for Postgraduate, Cairo University, Zayed City 12588, Egypt; drsameheltayer@yahoo.com

In the original publication [1], there was a mistake in the legend for Figure 5. The original caption did not clearly distinguish between TEM and SEM images and failed to specify that some SEM images represented the same sample at different magnification levels. The correct legend appears below.

**Figure 5.** Microscopic characterization of silver-protein nanocomposites: (**A**–**C**). TEM images of three different silver-protein nanocomposite formulations showing distinct morphological features at 100 nm scale; (**A**–**C**), SEM images of the same silver nanoparticle sample imaged at progressive magnification levels (1 μm, 500 nm, and 500 nm respectively) to demonstrate the hierarchical surface morphology and aggregation patterns of silver nanoparticles. The sequential magnification allows visualization of both the overall distribution and the detailed surface features of the same nanostructure. The all SEM images for silver-mung bean seed protein nanocomposite (Ag-MNP) (Supplementary Figure S1) and silver-fenugreek seed protein nanocomposite (Ag-FNP). (Supplementary Figure S2) SEM images in supplementary file attached.

The original publication did not provide adequate clarification regarding the TEM image interpretation in Figure 5B and the nature of background matrix textures in nanocomposite materials. The correction that has been made to Results, Section 3, Paragraph 3, appears below:

In Figure 5B, the TEM image represents transmission electron microscopy of silver-protein nanocomposite material. The background matrix textures visible in the image are characteristic of the protein matrix in which the silver nanoparticles are embedded. In transmission electron microscopy of nanocomposites, such consistent background patterns are expected due to the uniform protein matrix structure and do not indicate image manipulation. The apparent similarities in nanoparticle regions result from our controlled synthesis protocol, which was specifically designed to produce particles with uniform size distribution and morphology using highly controlled synthesis methods.

To enhance transparency and reproducibility, additional original SEM images of both silver-mung bean seed protein nanocomposite (Ag-MNP) and silver-fenugreek seed protein nanocomposite (Ag-FNP) samples have been included in supplementary materials as Figures S1 and S2, respectively. These images provide further evidence supporting our findings regarding the morphological characterization of the nanocomposites and demonstrate the reproducibility of our synthesis methods.

In section Supplementary Materials, the following information should be included:

**Supplementary Materials:** The following are available online at https://www.mdpi.com/article/10.3390/nano11113006/s1, Supplementary Figure S1. Silver-mung bean seed protein nanocomposite (Ag-MNP) SEM images; Supplementary Figure S2. Silver-fenugreek seed protein nanocomposite (Ag-FNP) SEM images; Supplementary Figure S3. The specific surface area measured with the Brunauer–Emmett–Teller isotherm (BET) of (A) FNP; (B) Ag-FNP; (C) MNP; (D) Ag-MNP; Supplementary Figure S4. The isotherm curve of (A) FNP; (B) Ag-FNP; (C) MNP; (D) Ag-MNP; Supplementary Figure S5. Antibacterial activity of silver mung bean protein nanocomposite (Ag-MNP), and silver fenugreek protein nanocomposite (Ag-FNP) against Gram-positive and Gram-negative bacteria.

The authors state that the scientific conclusions are unaffected. This correction was approved by the Academic Editor. The original publication has also been updated.
