# Supplementary material for: Silver-Protein Nanocomposites as Antimicrobial Agents
Source: Nanomaterials (Basel). 2021 Nov 9;11(11):3006. doi: 10.3390/nano11113006 (PMC8617916; doi:10.3390/nano11113006)
Supplement: Supplementary file 1 [file nanomaterials-11-03006-s001.zip › nanomaterials-1416877-supplementary.pdf]

Supplementary Materials:

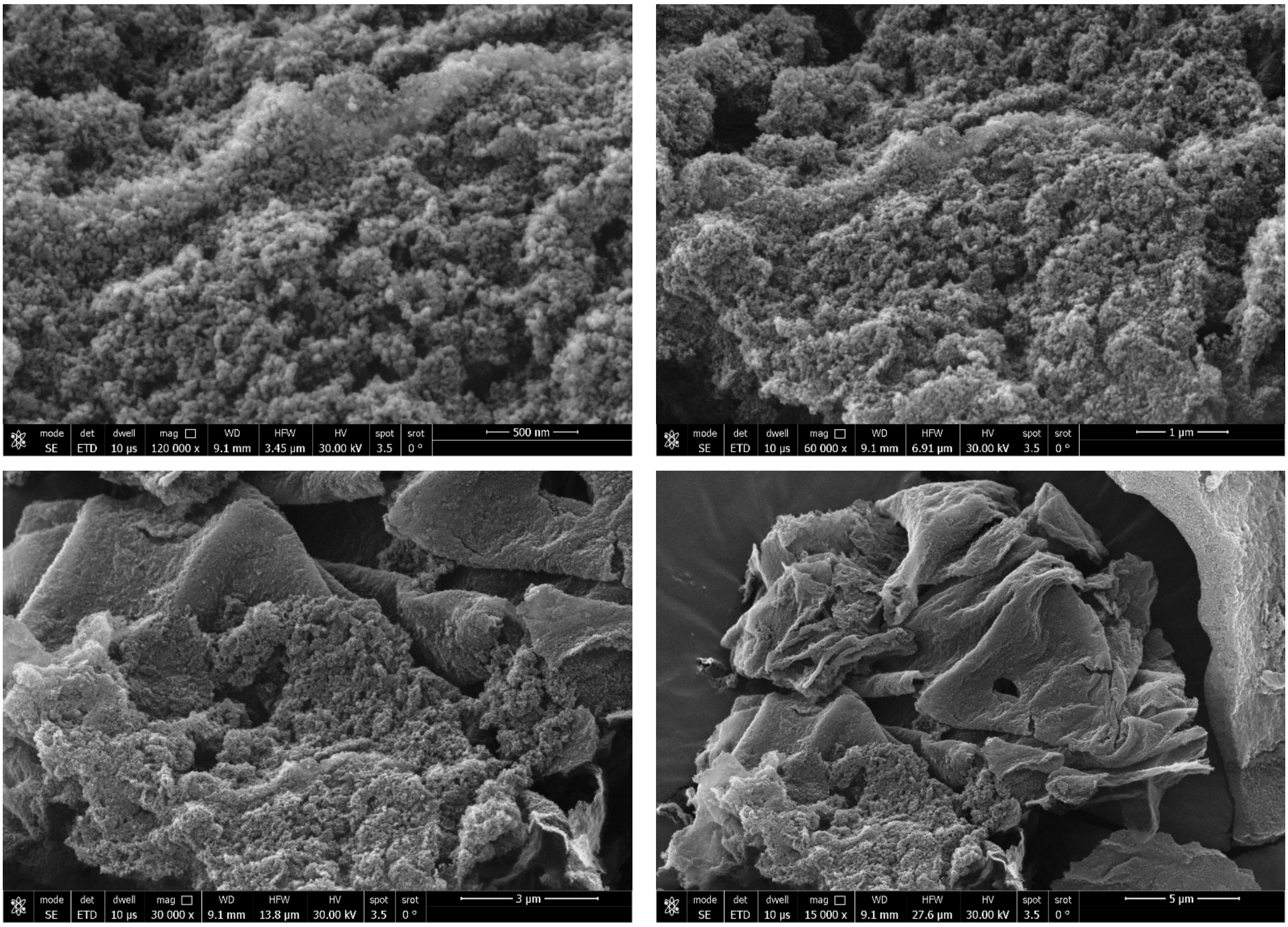

Supplementary Figure S1. Silver-mung bean seed protein nanocomposite (Ag-MNP) SEM images.

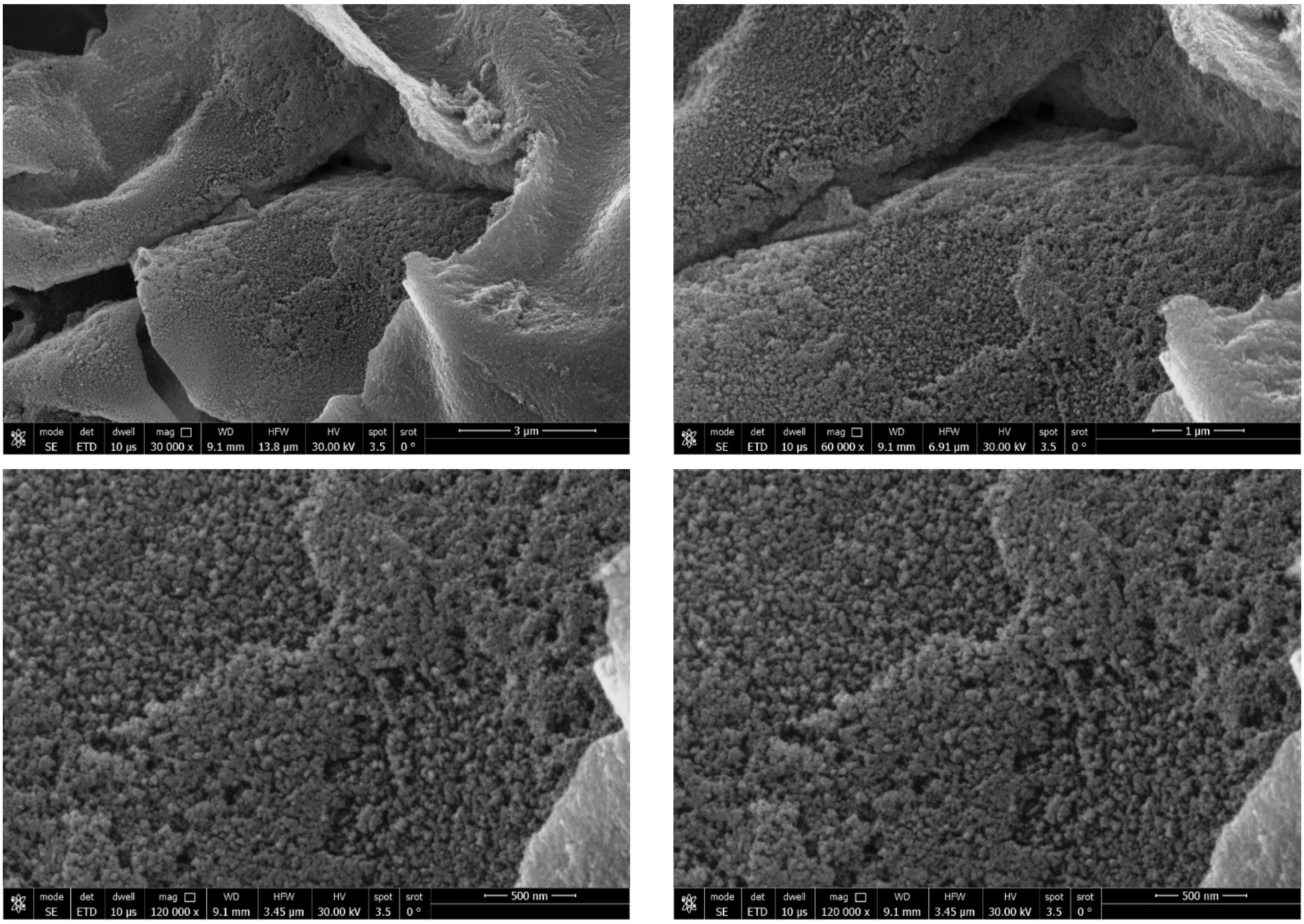

Supplementary Figure S2. Silver-fenugreek seed protein nanocomposite (Ag-FNP) SEM images.

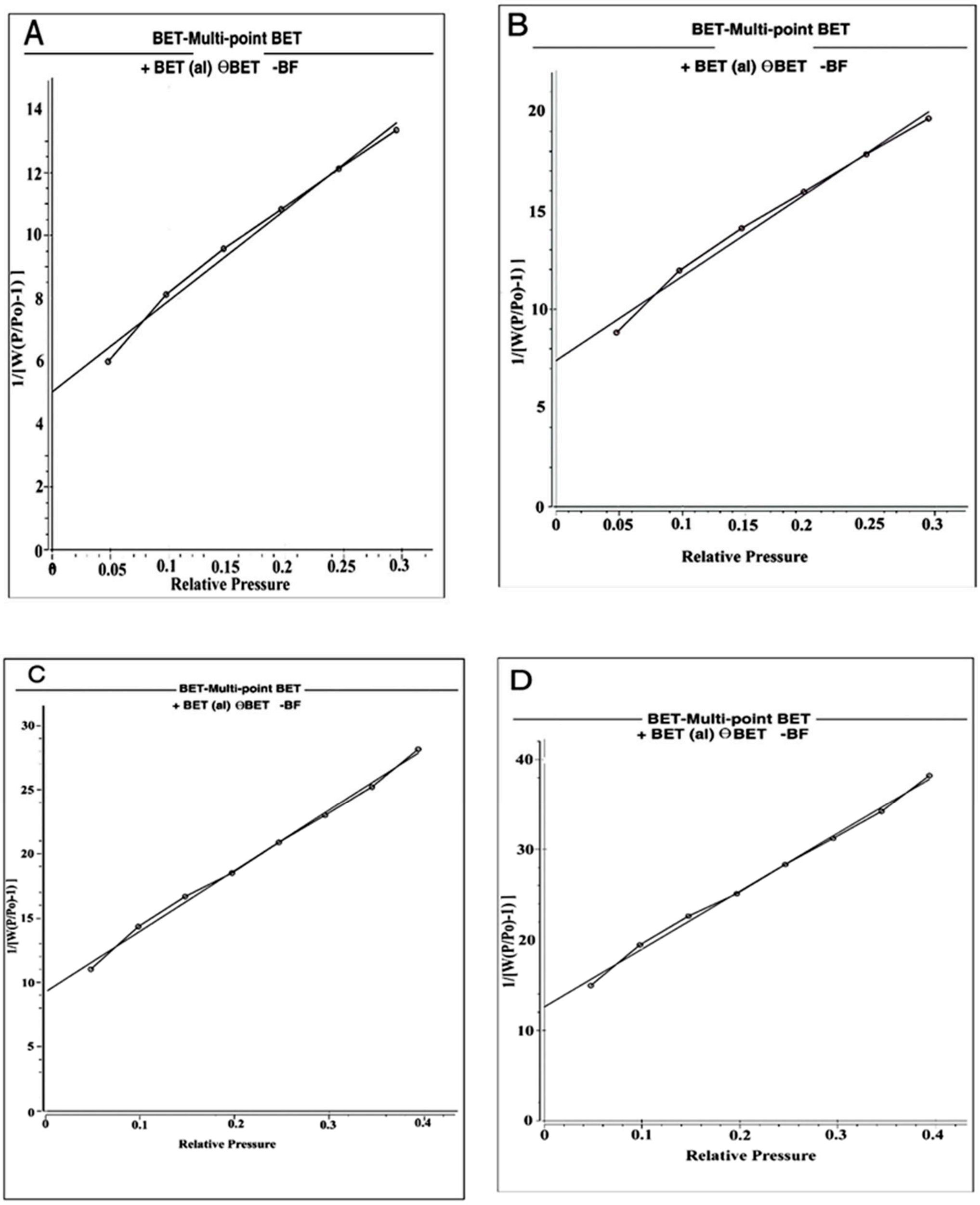

Supplementary Figure S3. The specific surface area measured with the Brunauer–Emmett–Teller isotherm (BET) of (**A**) FNP; (**B**) Ag-FNP; (**C**) MNP; (**D**) Ag-MNP.

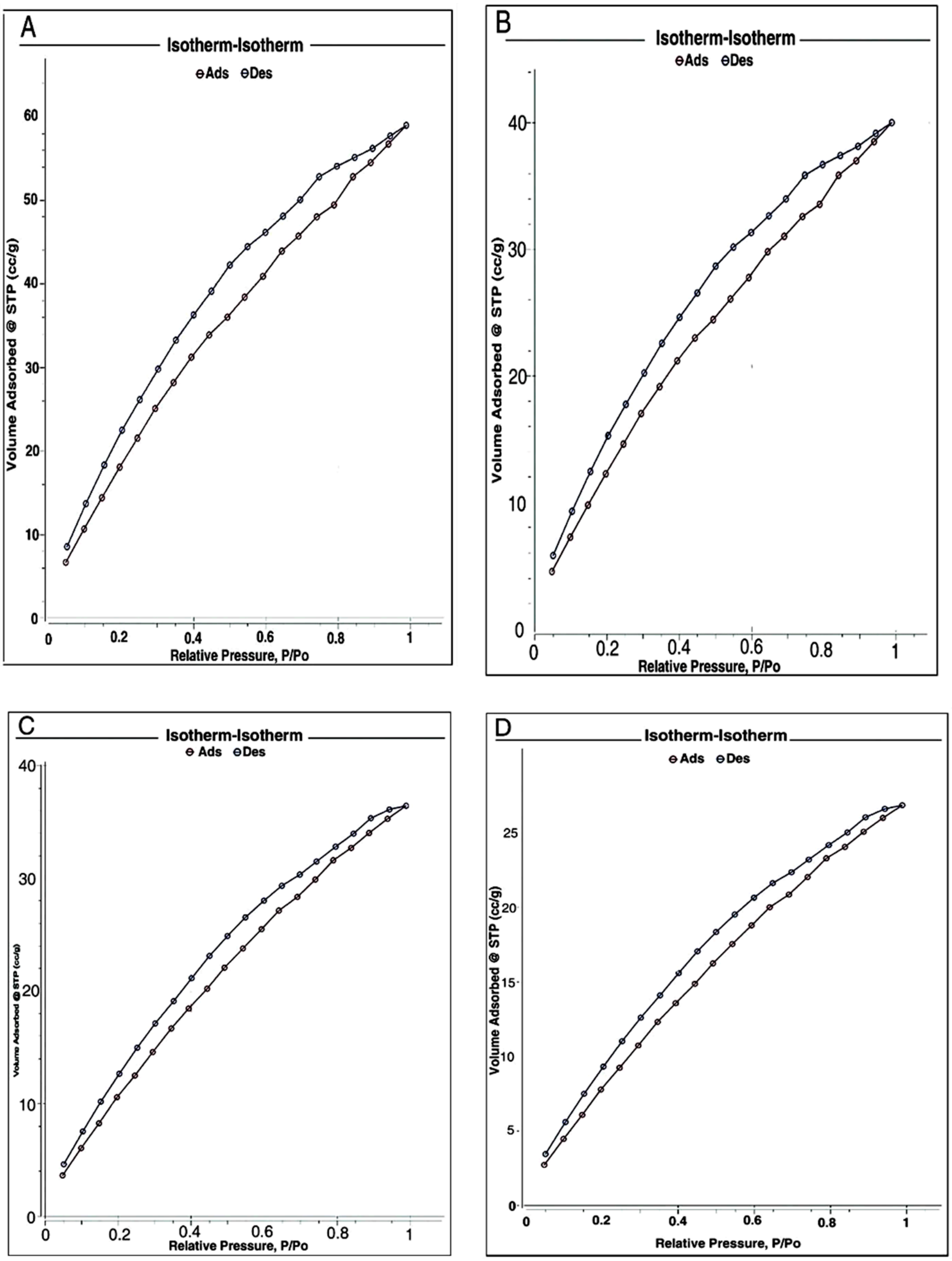

Supplementary Figure S4. The isotherm curve of (**A**) FNP; (**B**) Ag-FNP; (**C**) MNP; (**D**) Ag-MNP.

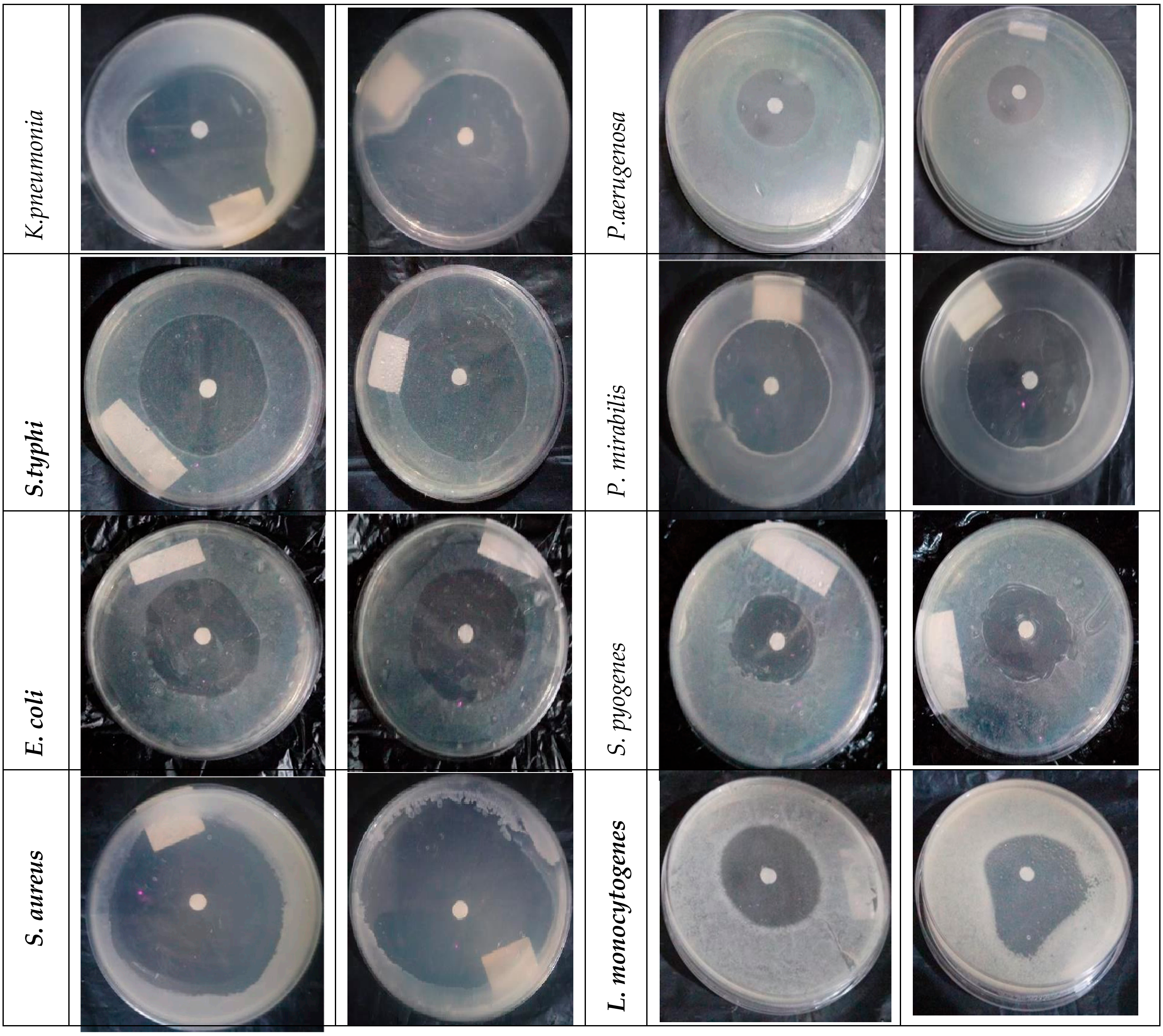

Supplementary Figure S5. Antibacterial activity of silver mung bean protein nanocomposite (Ag-MNP), and silver fenugreek protein nanocomposite (Ag-FNP) against Gram-positive and Gram-negative bacteria.
